# Optimizing diastolic pressure gradient assessment

**DOI:** 10.1007/s00392-020-01641-w

**Published:** 2020-05-11

**Authors:** Aristomenis Manouras, Jonas Johnson, Lars H Lund, Anikó Ilona Nagy

**Affiliations:** 1 Department of Medicine, Karolinska Institute, Solna, Stockholm, Sweden; 2 Centre for Fetal Medicine Department of Obstetrics and Gynecology, Karolinska University Hospital, Stockholm, Sweden; 3 Theme of Heart and Vessels, Karolinska University Hospital, Stockholm, Sweden; 4 Heart and Vascular Center, Semmelweis University, 68. Városmajor u., Budapest, 1026 Hungary

**Keywords:** Diastolic pressure gradient, Pulmonary wedge pressure, *Y*-descent, V-wave, Prognosis, Heart failure

## Abstract

**Aims:**

The diastolic pressure gradient (DPG) has been proposed as a marker pulmonary vascular disease in the setting of left heart failure (HF). However, its diagnostic utility is compromised by the high prevalence of physiologically incompatible negative values (DPG_NEG_) and the contradictory evidence on its prognostic value. Pressure pulsatility impacts on DPG measurements, thus conceivably, pulmonary artery wedge pressure (PAWP) measurements insusceptible to the oscillatory effect of the V-wave might yield a more reliable DPG assessment. We set out to investigate how the instantaneous PAWP at the trough of the *Y*-descent (PAWP_*Y*_) influences the prevalence of DPG_NEG_ and the prognostic value of the resultant DPG_*Y*_.

**Methods:**

Hundred and fifty-three consecutive HF patients referred for right heart catheterisation were enrolled prospectively. DPG, as currently recommended, was calculated. Subsequently, PAWP_*Y*_ was measured and the corresponding DPG_*Y*_ was calculated.

**Results:**

DPG_*Y*_ yielded higher values (median, IQR: 3.2, 0.6–5.7 mmHg) than DPG (median, IQR: 0.9, − 1.7–3.8 mmHg); *p* < 0.001. Conventional DPG was negative in 45% of the patients whereas DPG_*Y*_ in only 15%. During follow-up (22 ± 14 months) 58 patients have undergone heart-transplantation or died. The predictive ability of DPG_*Y*_ ≥ 6 mmHg for the above defined end-point events was significant [HR 2.1; *p* = 0.007] and independent of resting mean pulmonary artery pressure (PAP_*M*_). In contrast, conventional DPG did not comprise significant prognostic value following adjustment for PAP_*M*_.

**Conclusion:**

Instantaneous pressures at the trough of *Y*-descent yield significantly fewer DPG_NEG_ than conventional DPG and entail superior prognostic value in HF patients with and without PH.

**Graphic abstract:**

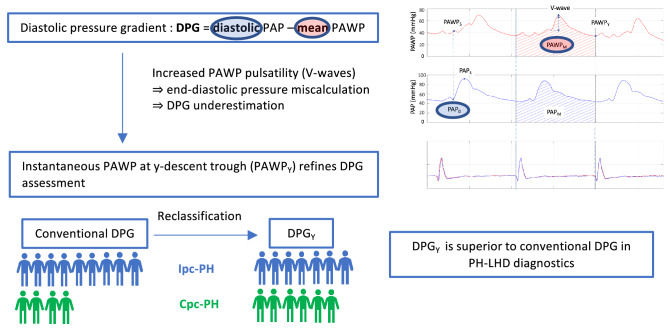

**Electronic supplementary material:**

The online version of this article (10.1007/s00392-020-01641-w) contains supplementary material, which is available to authorized users.

## Introduction

Secondary pulmonary vascular disease is a common complication of heart failure (HF). In addition to a passive backward transmission of elevated filling pressures to the pulmonary venous system, structural and functional alterations of the pre-capillary pulmonary vasculature may occur resulting in further increase of the right ventricular afterload, associated with poor prognosis [1–6]. In pulmonary hypertension due to left heart disease (PH-LHD), the diastolic pressure gradient (DPG), i.e. the difference between the pulmonary artery diastolic pressure (PAP_*D*_) and the mean pulmonary artery wedge pressure (PAWP_*M*_), has been proposed as a specific marker of pre-capillary involvement [7], which is an important part of the evaluation for transplant candidacy. Nevertheless, the initially demonstrated prognostic superiority of DPG over traditional markers of precapillary pulmonary vascular changes [8–10] was not corroborated in subsequent large-scale studies [11–15], raising concerns about the utility of the DPG. Although indeed thought provoking, the aforementioned discrepancy may not infer that the overall concept of DPG is invalid; it might rather reflect important and potentially amendable methodological inaccuracies in the DPG calculation.

While the PAP_*D*_ constitutes an instantaneous late diastolic event, the PAWP_*M*_ encompasses both steady and pulsatile components integrated throughout the cardiac cycle. Not surprisingly, PAWP_*M*_ often overestimates the diastolic left atrial pressure (LAP), particularly in the occurrence of augmented pulsatility during the V-wave [16]. The subsequent DPG underestimation, consistent with the high prevalence of negative DPG values (DPG_NEG_) [11, 16, 17], might also partly stand for the ambiguity regarding the DPG’s prognostic value [11, 12]. In our previous work we demonstrated that negative DPG values indeed are in large part attributable to the presence of large V-waves. Thus, it is conceivable that pressure measurements more representative of the diastolic LAP that obviate the effect of systolic V-waves would be preferential for achieving a more reliable DPG assessment [18].

**Fig. 1:**
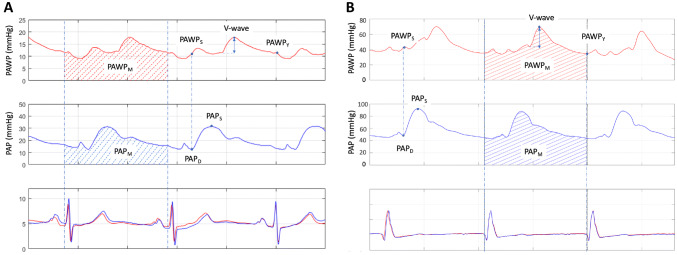
Pulmonary artery wedge pressure measurements in a patient with normal (**a**) and another with high V-wave (**b**). The top panels shows the pulmonary artery wedge pressure (PAWP), the middle panels the pulmonary artery pressure (PAP) waveform, the bottom panels the corresponding ECG traces for both measurements (ECG of the PAWP curve in red, ECG of the PAP curve in blue). First the two pressure waveforms were synchronized, using the ECG, so that simultaneous pressure waveforms were achieved over at least three heart cycles. On the PAWP waveform the following instantaneous pressure values were measured: peak of the V-wave, PAWP at the trough of the *Y*-descent (PAWP_*Y*_), PAWP at the time point that is simultaneous with PAP_*D*_ (PAWP_*S*_). Absolute V-wave was defined as the pressure difference between the beginning of the ascending limb of the V-wave and the peak V-wave pressure. On the PAP waveform the following instantaneous pressure values were measured: peak of the ascending limb of the PAP curve (PAP_*S*_) and the point at the end of diastole (PAP_*D*_). In addition, the software provided automated calculation of mean PAP (PAP_*M*_) and PAWP (PAWP_*M*_) by integrating the PAP or PAWP, respectively, over the entire cardiac cycle. PAWP values for panel **a** PAWPmean = 13.5 mm Hg PAWP_*Y*_ = 12 mm Hg; panel **b** PAWPmean = 40 mm Hg, PAWP_*Y*_ = 36 mm Hg

In an early study, Braunwald and colleagues demonstrated that the instantaneous c-wave pressure on the PAWP curve provided a better estimate of the left ventricular end-diastolic pressures (LVEDP) compared to PAWP_*M*_ [19]. However, the c-wave is often absent or difficult to find. Another approach to approximate the diastolic PAWP is to measure the mean A-wave, i.e. the mean of the highest and lowest A-wave pressure. An inherent major limitation of both of the aforementioned methods is that patients with atrial arrhythmias lack an A-wave and consequently a c-wave'; therefore, in a significant proportion of patients these measurements are not feasible. This limitation is overcome by the method, recently proposed by Wright and colleagues, namely to use the onset of the QRS complex to approximate the end diastolic PAWP [20]. This measurement is attainable in all patients; on the other hand, due to the time delay between the left atrial and PAW pressure together with the delay between depolarization and contraction, the suggested method will not actually capture end diastolic PAWP [21]. Another group suggested that measuring the pressure at the base of the descending branch of the V-wave (*Y*-descent) might provide a more representative diastolic LAP value for the purpose of DPG calculation (Fig. 1) [22]. We hypothesized that this latter approach might be less susceptible to the distortive V-wave pulsatility, and might provide a physiologically sound, feasible and more robust DPG assessment. Thus, we set out to (1) investigate the influence of this measurement on the DPG, in particular the occurrence of DPG_NEG_ values; and (2) to assess the prognostic value of DPG based on the suggested alternative PAWP measurement method in LHD patients.

## Materials and methods

### Study population

Two hundred and twenty patients referred for right heart catheterization (RHC) at the Karolinska University Hospital for hemodynamic assessment because of known or suspected HF, between February 2014 and June 2017, were enrolled prospectively. Of them 11 patients who previously underwent cardiac transplantation (HX) were excluded. By the results of catheterization, 29 patients turned out to have an underlying disease other than primary left heart myocardial disease (pulmonary arterial hypertension, *n* = 15; constrictive pericarditis, *n* = 14) and were, therefore, excluded from further analysis. In addition, three patients with ARVD, ten patients with significant valvular disease (five severe mitral regurgitation (MR) and five with severe tricuspid regurgitation) and three patients with poor echocardiographic image quality were also excluded. In 11 cases the RHC pressure tracings were judged to have inadequate quality, these were not included in the final analysis (Figure S1). Patients were followed up during a mean period of 558 days [IQ range: 295–950]. The end-point of the study was the combined outcome of death or HX/left ventricular assist device (LVAD) implantation.

The study conformed to the Declaration of Helsinki and was approved by local ethics committee. All participants provided written informed consent.

### Echocardiography

All subjects underwent transthoracic echocardiography within 1 h prior to RHC, using an E9 system (GE Ultrasound, Horten, Norway) equipped with a 2.5-MHz matrix array transducer, in keeping with current guidelines [23].

### Catheterization

During RHC all patients were in haemodynamically stable condition and euvolemic status. RHC was performed in using a 6F balloon-tipped fluid-filled Swan-Ganz catheter (Edwards Lifesciences, Irvine, CA, USA) through the jugular vein access. Mean right atrial pressure, pulmonary artery pressures, PAWP and right ventricular systolic pressure were recorded under fluoroscopy after calibration with the zero-level set at the mid-thoracic line. Measurements were recorded at end-expirium during spontaneous breathing and stored in a connected haemodynamic recorder (Xper Information Management, Philips Medical Systems, The Netherlands). Cardiac output (CO) was measured using the Fick’s principle. The oxygen consumption was measured breath-by-breath (Jaeger Oxycon Pro, VIASYS™ Healthcare, Palm springs, CA, USA) in ml/min. Arterio-venous oxygen difference was calculated from oxygen concentration in arterial and mixed venous blood from the pulmonary artery. In ten cases thermodilution was employed.

### Exercise protocol

Following the assessment of resting haemodynamics, patients with normal PAWP_*M*_ at rest (≤ 15 mmHg) and without significantly elevated PAP_*M*_ or with clinical suspicion of HFpEF performed supine cycle ergometry. Furthermore, patients with HFrEF also underwent ergometry as part of the haemodynamic evaluation in our lab. Patients cycled at 60 rpm starting at a 20-W workload and increasing by 10-W increments in 1-min stages to maximum tolerated levels. PAWP_*M*_ was determined at peak exercise. Prior studies in normal controls have shown that peak PAWP_*M*_ during supine exercise is < 20–23 mmHg [21, 24–26]. In our study, PAWP_*M*_ ≥ 23 mmHg during peak exercise denoted an abnormal PAWP_*M*_ response.

### Off-line analysis of RHC waveforms

PAWP and PAP waveforms were individually reviewed and those of good quality for analysis (*n* = 153) exported from the haemodynamic recorder and then imported into MATLAB software (R2018b, MathWorks, MA, USA). This system allowed simultaneous display of both waveforms along with the corresponding ECG traces. First the ECGs of the two recordings were synchronised manually so that despite non-beat-to-beat synchronous measurements, temporal synchronisation was achieved.

From the PAP recordings, the peak of the ascending limb of the PAP curve (PAP_*S*_) as well as the end diastolic pressure (PAP_*D*_) was identified and marked manually, following which the software provided an automated calculation of *PAP*_*S*_ and PAP_*D*._ Subsequently mean PAP (PAP_*M*_) was calculated by integration of PAP over the entire cardiac cycle. Similarly, on the PAWP waveform the point signifying the peak of the V-wave and the trough of the *Y*-descent (PAWP_*Y*_) were marked, as well as the time point at which the ECG-synchronized PAP_*D*_ was obtained (PAWP_*S*_) (Fig. 1). Additionally, automated integration of the PAWP waveform over the entire cardiac cycle was also obtained (PAWP_*M*_). All pressure measurements were averaged from a minimum of three heart cycles at end-expiration. Importantly, in order to ensure the uniformity of data acquisition and analysis the same investigator (AM) participated in the majority of RHC procedures and performed the analysis of all waveforms. For the analysis, data were anonymized; thus all analysis was performed in a blinded fashion. Large V-waves were defined as the peak V-wave exceeding the PAWP_*M*_ by ≥ 10 mmHg [27]. The reproducibility of the instantaneous PAWP measurements were determined in ten randomly selected patients.

### Statistical analysis

The IBM SPSS statistics version 23.0 was used. Normality was tested by the Shapiro–Wilk test. Continuous variables were expressed as median and interquartile ranges, and categorical variables as absolute values and percentage. The Wilcoxon test and Mann–Whitney *U* test were used for matched samples and comparisons between independent groups, respectively. Correlations were tested by Spearman test. For comparison of differently obtained PAWP measurements as well as the derived DPG values Bland–Altman analysis was used. All tests were performed at 95% confidence intervals. A *p* value of < 0.05 was considered statistically significant. The predictive value of the differently obtained DPG values for the combined outcome of death or heart-transplantation (HX) was tested using a time to event analysis with univariate and multivariable Cox proportional hazards models and Kaplan–Meier non-parametric test and compared employing a log-rank test. The proportional hazards assumption was tested for all analyses.

## Results

### Demographics

Demographic data are provided in Table 1. Recordings of 153 patients were analysed (age 60 [50–74] years; 43% females), who all fulfilled the diagnostic criteria of HF, having elevated PAWP_*M*_ (> 15 mmHg) at rest or during exercise testing (≥ 23 mmHg). 51% had preserved ejection fraction (EF ≥ 50%). 88 (57%) patients had elevated PAP_*M*_ (≥ 25 mmHg) at rest, whereas all patients demonstrated elevated PAP_*M*_ (≥ 35 mmHg) and PAWP_*M*_ (> 23 mmHg) upon exercise.

**Table 1:** Demographic data

	All patients (153)	HFpEF (78)	HFrEF (75)
Demographics			
Age	62 (50–74)	71 (60–78)**	56 (46–63)
Female (%)	43	63	25
BMI (kg/m^2^)	26.7 (22.8–29.7)	26.8 (22.8–29.7)	25.7 (22.7–29.4)
HT (%)	61	62	49
DM (%)	14	13	15
IHD (%)	23	13	33
HFpEF (%)	51%		
HR (bpm)	68 (60–78)	68 (60–78)	68 (60–79)
Non-sinus rhythm (%)	30	32	28
Functional class			
NYHA I	7%	12%	1%
NYHA II	18%	22%	14%
NYHA III–IV	75%	66%	85%
Medication			
Diuretics	80%	71%	88%
ACEi/ARB	63%	46%	81%
Beta blockers	79%	66%	92%
CCA	18%	22%	13%
MRA	54%	36%	74%
Echo data			
EF	53 (26–63)	61 (56–65)**	27 (20–42)
LVEDD (mm)	50 (40–60)	50 (40–50)*	60 (50–75)
LVESD (mm)	37 (28–56)	29 (25–33)**	56 (45–65)
LA-ESVi (ml/m^2^)	45 (35–62)	42 (34–55)*	49 (38–68)
RVEDD (mm)	41 (35–47)	41 (34–45)	42 (36–48)
TAPSE (mm)	15 (12–20)	17 (13–24)**	14 (11–17)
> gr II MR (%)	9	7	11
Biochemical data			
NT-proBNP (ng/l)	1940 (605–2965)	1100 (295–2730)*	2270 (1250–3590)
Hb (g/ml)	132 (118–145)	125 (114–142)*	137 (124–147)
Creatinine (μmol/l)	93 (71–118)	80 (65–110)**	98 (84–127)

Continuous values are expressed as median followed by interquartile ranges in brackets

*BMI* body mass index, *HT* hypertension, *DM* diabetes mellitus, *IHD* ischaemic heart disease, *HFpEF* heart failure with preserved ejection fraction, *HR* heart rate, *bpm* beats per minute, *NYHA* New York Heart Association functional class, *ACEi* angiotensin-convertase inhibitor, *ARB* angiotensin receptor blocker, *CCA* calcium channel blocker, *MRA* mineralocorticoid receptor antagonist, *EF* ejection fraction, *LVEDD* left ventricular end-diastolic diameter, *LVESD* left ventricular end-systolic diameter, *LA-ESVi* left atrial end-systolic volume indexed to BSA, *RVEDD* right ventricular end-diastolic diameter, *TAPSE* tricuspid annulus plane systolic excursion, *Hb* haemoglobin

*p < 0.05; **p < 0.001

At the time of enrollment all patients were symptomatic. Ischemic cardiomyopathy was the cause of HF in 20 cases, idiopathic dilated cardiomyopathy in 53, restrictive cardiomyopathy of various origin in 21 (amyloidosis: 5, sarcoidosis: 1, hypertrophic: 5, other: 10), and viral myocarditis in one case, with the rest being of multifactorial origin. Moderate MR was present 13 and mild MR in 135 patients; in all cases the MR was functional.

### Methodological validation

PAWP_*M*_ showed strong correlation with both PAWP_*Y*_ (*r* = 0.94, *p* < 0.001) and PAWP_*S*_ (*r* = 0.93, *p* < 0.001) measurements. However, as shown in Table 2, PAWP_*Y*_ yielded significantly lower pressures as compared to PAWP_*M*_ [median bias: − 2.2 (− 3.9 to − 1.2)], with a hardly discernible underestimation of PAWP_*S*_ [PAWP_*Y*_–PAWP_*S*_: median bias − 0.3 (− 1.5 to 0.5)].

**Table 2:** Haemodynamic characteristics of the patients classified by EF group

	All patients (153)	HFpEF (78)	HFrEF (75)	Frequency of negative values
PAP_*M*_ (mmHg)	27 (22 to 34)	27 (22 to 36)	27 (22 to 32)	
PAP_*D*_ (mmHg)	18 (14 to 23)	18 (13 to 23)	19 (14 to 24)	
PAWP_*M*_ (mmHg)	17.3 (13.3 to 23.7)	16.5 (13.3 to 21.5)	18.8 (13.3 to 24.4)	
Peak V-wave (mmHg)	22.8 (16.5 to 31.1)	22.8 (16.5 to 31.2)	23.2 (16.3 to 31.0)	
Large V-waves	27 (18%)	19 (24%)	8 (10.7%)	
CI (l/min/m^2^)	2.3 (1.9 to 2.7)	2.5* (2.0 to 3.1)	2.2 (1.8 to 2.4)	
DPG (mmHg)	0.9 (− 1.7 to 3.8)	0.9 (− 1.4 to 4.3)	0.9 (− 1.8 to 3.2)	68 (45%)
TPG (mmHg)	10 (7 to 14)	11* (8 to 15)	8 (5 to 12)	
PVR (WU)	2.2 (1.4 to 3.2)	2.5* (1.5 to 3.5)	1.9 (1.1 to 3.0)	
PAWP_*Y*_ (mmHg)	14.2 (11.4 to 18.9)	13.6 (11.0 to 18.2)	16.2 (11.8 to 19.6)	
PAWP_*S*_ (mmHg)	15.2 (11.9 to 20)	13.8* (11.6 to 18.2)	15.9 (12.1 to 21.4)	
Bias [PAWP_*Y*_–PAWP_*M*_] (mmHg)	− 2.2 (− 3.9 to − 1.2)			
Bias [PAWP_*S*_–PAWP_*M*_] (mmHg)	− 1.9 (− 3.4 to − 0.5)			
Bias [PAWP_*Y*_–PAWP_*S*_] (mmHg)	− 0.3 (− 1.5 to 0.5)			
DPG_*Y*_ (mmHg)	3.7 (1.5 to 5.7)	4.1 (1.8 to 5.9)	3.2 (1.0 to 5.6)	23 (15%)
DPG_*S*_ (mmHg)	3.2 (0.6 to 5.7)	3.6 (1.2 to 5.8)	2.4 (− 0.1 to 5.7)	30 (20%)

Continuous variables are expressed as median followed by interquartile ranges in brackets. Frequencies are expressed as number of patients followed by percentages in brackets

*HFpEF* heart failure with preserved ejection fraction, *HFrEF* heart failure with reserved ejection fraction, *PAP*_*M*_ pulmonary artery mean pressure, *PAP*_*D*_ pulmonary artery diastolic pressure, *PAWP*_*M*_ mean pulmonary artery wedge pressure, *CI* cardiac index, *DPG* diastolic pressure gradient, *TPG* trans-pulmonary gradient, *PVR* pulmonary vascular resistance, *PAWP*_*Y*_ pulmonary artery wedge pressure measured at the trough of the *Y*-descent, *PAWP*_*S*_ pulmonary artery wedge pressure measured simultaneously with the time-point of PAP_*D*_, *DPG*_*Y*_ DPG derived from PAWP_*Y*_, *DPG*_*S*_ DPG derived from PAWP_*S*_

*Signifies statistically significant difference (*p* < 0.05) between HFpEF and HFrEF

**Fig. 2:**
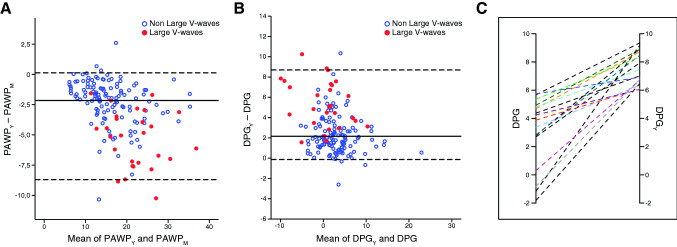
Bland–Altman analysis of instantaneous (PAWP_*Y*_) vs integrated mean pulmonary artery wedge pressure (PAWP_*M*_) (**a**) and the derived DPG_*Y*_ and DPG values (**b**). Median values and 97.5% and 2.5% CI are presented. **c** Changes of DPG in patients reclassified from normal by conventional DPG to pathological by DPG_*Y*_. Axes represent DPG values in mmHg. *PAWP*_*M*_ mean pulmonary artery wedge pressure, *PAWP*_*Y*_ PAWP measured at the trough of the *Y*-descent, *DPG* diastolic pressure gradient, *DPG*_*Y*_ DPG calculated using PAWP_*Y*_, *CI* confidence interval

Importantly, the discrepancy between the PAWP_*M*_ and the two instantaneous PAWP measurements was accentuated with increasing pressure and/or at the presence of large V-waves, whereas the degree of concordance between PAWP_*Y*_ and PAWP_*S*_ was kept similar along the whole pressure range and was independent of large V-waves (Fig. 2).

Notably, the relation of all three PAWP measurements with either PAP_*M*_ or PAP_*D*_ was essentially identical (correlation of PAP_*M*_ with PAWP_*M*_ *r* = 0.83, with PAWP_*Y*_ *r* = 0.82, with PAWP_*S*_ *r* = 0.75; correlation of PAP_*D*_ with PAWP_*M*_ *r* = 0.81, with PAWP_*Y*_ *r* = 0.83, with PAWP_*S*_ *r* = 0.81; *p* < 0.001 for all).

Reproducibility measures of the instantaneous PAWP measurements were excellent, with an intra-observer intra-class correlation coefficient (ICC) of 0.98 and inter-observer ICC of 0.97 for PAWP_Y_ measurement.

### Haemodynamic implications on DPG

The DPG_*Y*_ values derived from PAWP_*Y*_ were significantly higher than conventional DPG calculated using PAWP_*M*_ [DPG_*Y*_ = 3.2 (0.6 to 5.7) vs. DPG = 0.9 (− 1.7 to 3.8) mmHg, *p* < 0.001]. Similarly, DPG_*S*_ values [3.7 (1.5 to 5.7) mmHg] were significantly higher compared to the conventional DPG [*p* < 0.001] (Table 2).

Accordingly, among DPG_*Y*_ and DPG_*S*_ there was a significantly lower prevalence of DPG_NEG_ (15% and 20%, respectively) compared to conventional DPG (45%).

Large V-waves (peak V-wave amplitude—PAWP_*M*_ ≥ 10 mmHg) were present in 27 (18%) patients, of whom essentially all displayed DPG_NEG_ [DPG = − 1.6; (− 4.7 to − 0.1) mmHg], with a significantly lower median value compared to the group without large V-waves [DPG = 1.4 (− 1.2 to 4.0) mmHg, *p* < 0.001]. In contrast, DPG_*Y*_ values did not differ between the two V-wave groups [DPG_*Y*_: 3.9 (0.1 to 5.4) mmHg, in large V-wave group; DPG_*Y*_: 3.7 (1.5 to 5.7) mmHg, in non-large V-wave group; *p* > 0.05 in both cases]. Accordingly, the V-wave amplitude demonstrated a significant inverse association with the conventional DPG (*r* = − 0.45, *p* < 0.001), but not with the DPG_*Y*_ or DPG_*S*_ (*p* > 0.05, in both cases).

Finally, PAP_*D*_ exhibited a stronger association with DPG_*Y*_ (*r* = 0.57, *p* < 0.001), and DPG_*S*_ (*r* = 0.52, *p* < 0.001) compared to the corresponding relationship with the conventional DPG (*r* = 0.34, *p* < 0.001).

In the subgroup of patients with resting PAP_*M*_ ≥ 25 mmHg the DPG_*Y*_ was higher 4.9 (1.9 to 7.3) mmHg, compared to the group with PAP_*M*_ < 25 mmHg [2.6 (0.3 to 4.2) mmHg, (*p* < 0.001)]. In contrast, conventional DPG did not differ between the two subgroups (PH-group: 1 [− 1.7 to 4.6] vs. non-PH-group 0.9 [− 1.6 to 3.2] mmHg; *p* = 0.3). The corresponding values for PVR for the two subgroups were 2.6 [1.7 to 4.8] vs. 1.8 [1.2 to 2.3] WU (*p* < 0.001).

### The prognostic value of DPG_*Y*_ in HF patients

**Fig. 3:**
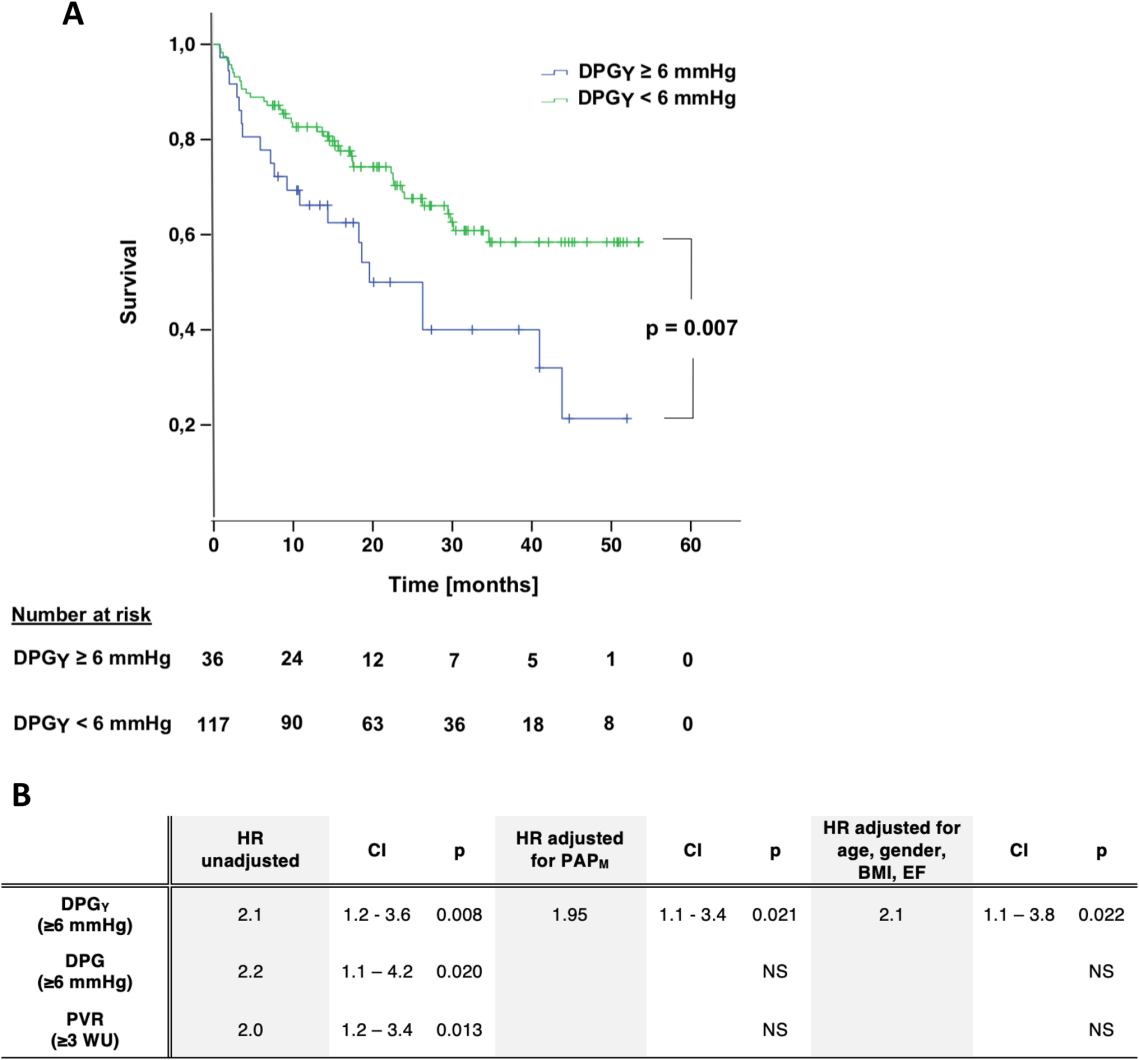
Prognostic value of DPG_*Y*_ in heart failure patients. **a** Kaplan–Meyer curve showing survival of patients with elevated and normal DPG_*Y*_, using cut-off value of 6 mmHg; **b** Cox proportional hazard models, classifying patients by DPG, DPG_*Y*_ and PVR, adjusted for clinical variables. *DPG* diastolic pressure gradient, *DPG*_*Y*_ DPG calculated using PAWP measured at the trough of the *Y*-descent, *HR* hazard ratio, *CI* confidence interval, *PAP*_*M*_ mean pulmonary artery pressure, *BMI* body mass index, *EF* ejection fraction, *PVR* pulmonary vascular resistance, *WU* wood units

In total, 58 events (28 deaths and 30 HX or LVAD implantations) occurred during the follow up period [median 558 days, IQ range: 295–950]. The prognostic ability of DPG and DPG_*Y*_ was assessed using Cox-regression analysis. DPG_*Y*_ was tested at different cut-off values of which the lowest that entailed significant prognostic value for the combined endpoint of death/HX/LVAD was at 6 mmHg (HR: 2.1; *p* = 0.007). Importantly, the prognostic ability of DPG_*Y*_ was independent of the presence of PH at resting RHC, as it remained significant when adjusted for resting PAP_*M*_ ≥ or < 25 mmHg (HR: 1.95; *p* = 0.021). Again, adjustment for clinical variables (gender, age, BMI and EF) did not impact on the prognostic strength of DPG_*Y*_ (HR: 2.1; *p* = 0.022) (Fig. 3). Conventional DPG was also tested for the most sensitive, lowest cut-off value, at which it entailed significant prognostic ability, which was identified at 6 mmHg (HR: 2.2; *p* = 0.02) (Figure S2). However, it did not remain predictive following adjustment for PAP_*M*_ ≥ or < 25 mmHg. Similarly, while pulmonary vascular resistance (PVR) at the cut-off value of 3 WU was a significant predictor of the combined outcome, it lost its prognostic ability following adjustment for PAP_*M*_.

Eighteen cases with normal DPG (< 6 mmHg) were reclassified as at increased risk for adverse events (≥ 6 mmHg) when using DPG_*Y*_. Importantly, of these patients a markedly higher proportion [9 out of the 16 reclassified cases (56%)] experienced an event during follow up, compared to 38% for the whole cohort.

In regard to the prognostic significance of DPG_NEG_, the incidence of death among DPG_NEG_ patients was still as high as 12%, only slightly lower than the corresponding value for the entire population (18%). Importantly, of the eight patients with DPG_NEG_ who died during follow-up, six cases were reclassified to positive when using DPG_*Y*_. Finally, comparison of the hemodynamic profiles of the patients with negative DPG or DPG_*Y*_ values (Table 3), demonstrated that negative DPG_*Y*_ was characterized by less pronounced hemodynamic alterations.

**Table 3:** Haemodynamic characteristics of patients with *A*, normal and pathological DPG_Y_ *B*, negative DPG_Y_ and negative DPG values

	*A*		*B*	
	DPG_*Y*_ < 6 (117)	DPG_*Y*_ ≥ 6 (36)	DPG_*Y*_ < 0 (23)	DPG ≥ 0 (68)
Age	62 (51–74)	63 (52–76)	56 (45–68)	60 (48–70)
BMI (kg/m^2^)	26 (23–29)	28 (24–32)	26 (22–29)	26 (23–30)
HR	67 (59–76)	71^✢^ (65–81)	63^✢^ (58–80)	68 (60–78)
EF	50 (26–63)	55 (25–63)	46 (25–60)	53 (29–64)
PAP_*M*_ (mmHg)	25 (21–32)	34^✢^ (31–44)	20^✢^ (16–30)	28 (22–33)
PAP_*D*_ (mmHg)	17 (13–21)	24^✢^ (21–33)	16 (12–25)	20 (15–25)
PAWP_*Y*_ (mmHg)	14 (11.3–18.6)	16.2 (12.4–22.1)	14.1 (10.6–21.9)	15.3 (12.3–19.9)
PAWP_*M*_ (mmHg)	17 (13–23)	19 (15–25)	16 (12–25)	20 (15–25)
Absolute V-wave (mmHg)	8 (4–11)	5 (3–9)	8 (6–13)	10 (7–16)
Prevalence of large V-waves	19%	14%	26%	31%
CI (l/min/m^2^)	2.3 (1.8–2.8)	2.3 (2.0–2.5)	4.7 (4.3–5.7)	4.6 (4.0–5.8)
TPG (mmHg)	9 (6–11)	15^✢^ (12–21)	4.3^✢^ (2.2–7.1)	7.2 (4.3–10.1)
PVR (WU)	1.9 (1.3–2.7)	3.2^✢^ (2.3–5.0)	0.9^✢^ (0.5–1.4)	1.6 (0.9–2.4)

*BMI* body mass index, *HR* heart rate, *EF* ejection fraction, *PAP*_*M*_ pulmonary artery mean pressure, *PAP*_*D*_ pulmonary artery diastolic pressure, *PAWP*_*M*_ mean pulmonary artery wedge pressure, *CI* cardiac index, *DPG* diastolic pressure gradient, *TPG* trans-pulmonary gradient, *PVR* pulmonary vascular resistance, *PAWP*_*Y*_ pulmonary artery wedge pressure measured at the trough of the *Y*-descent, *DPG*_*Y*_ DPG derived from PAWP_*Y*._

Interestingly, when stratifying patients according to their ejection fraction, both conventional DPG and DPG_*Y*_ remained prognostic in the pEF cohort (HR: 3.9, CI: 1.4–10.4, *p* = 0,007, HR: 3.9, CI: 1.6–9.6, *p* = 0.001, for DPG and DPG_*Y*_, respectively); however, it carried no prognostic information among patients with rEF (HR: 1.6, CI: 0.6–4.0, *p* = 0,36, HR: 1.4, CI: 0.7–2.8, *p* = 0.37, for DPG and DPG_*Y*_, respectively). This finding suggests potentially differential pulmonary vascular alterations within these two patient groups; however, due to the low case numbers after such division, caution should be exercised when interpreting this finding. Further studies are warranted to investigate the disparate diagnostic and prognostic utility of DPG in various HF cohorts.

## Discussion

The current study explores the validity of a novel approach for DPG assessment. We show that instantaneous LAP at the trough of the *Y*-descent evades the influence of pressure pulsatility and consequently substantially limits the occurrence of negative DPG values. Furthermore, we demonstrate that the resultant DPG_*Y*_ measurements have superior diagnostic ability compared to conventional DPG in discerning patients at risk for adverse events and entail significant prognostic information in HF patients both with elevated and with normal resting PAP.

It is common practice to deduce diastolic LV pressures from RHC-derived wedge pressures, as this approach is feasible and allows for comprehensive haemodynamic assessment. Nevertheless, early studies revealed that PAWP_*M*_ frequently overestimates the LVEDP, a discrepancy that has in large part been ascribed to the pulsatile PAWP elements [28, 29]. Rather than representing purely diastolic events, PAWP_*M*_ comprises an integration of systolic and diastolic LA pressures. It is thus conceivable that the phasic pressure oscillations that characterize the PAWP waveform by ensuing uneven pressure distribution might lead to overestimation of the diastolic atrial pressures when employing PAWP_*M*_. Hence, pressure measurements that are not directly affected by the V-waves are expected to provide more reliable estimation of the diastolic LAP. It has been shown that the pressure at the trough of *X*-descent yields improved concordance between the LAP and the LVEDP [29]. However, this approach suffers from an important inherent limitation, namely that it can only be employed in patients in sinus rhythm. In a recent investigation, instantaneous PAWP measurements at the onset of the QRS complex have been proposed for DPG calculation in order to attenuate the aforementioned methodological shortcomings of conventional DPG assessment [20]. This method, however, does not take into account the phase delay between the LAP and the PAWP, nor does it count with the electromechanical delay between depolarization and contraction. In reality the representation of the end-diastolic pressure on the PAWP waveform should occur 130–200 ms after the on the onset of the QRS complex on the surface ECG; thus this method may underestimate the PAWP and thus overestimate the DPG [21]. Indeed, Wright and co-workers did not find a mortality difference between the patient groups classified based on the meticulously calculated ECG-gated DPG values.

It has also been suggested that pressure measurements at the trough of *Y*-descent (PAWP_*Y*_), which coincide with the beginning of diastasis, might better represent LVEDP [22], a methodology that is easily applicable and feasible independently of the presence of supraventricular arrhythmias. In the present report we show that in contrast to PAWP_*M*_, PAWP_*Y*_ remains unaffected by the phasic pulsatile LAP components and yields systematically lower pressures, this difference being particularly evident in subjects with prominent V-waves. More importantly, the relationship of the obtained instantaneous PAWP_*Y*_ with the direct haemodynamic correlate of LAP remained unaltered as indicated by the PAP_*D*_ and the PAP_*M*_, demonstrating similar associations with PAPW_*M*_ and the corresponding PAWP_*Y*_.

Following its introduction, the DPG has gained primary importance in the PH-LHD diagnostics. However, the lack of consistency in the results of various studies on this metric has resulted in questioning its role in HP diagnostics. In fact, as defined in the yet unpublished ERS/ESC guidelines, the DPG is not any more recommended as a primary metric in the diagnostics of group 2 PH.

From a physiological perspective, the DPG ideally describes the functional state of pulmonary vasculature during cardiac diastasis, as it theoretically relies on diastolic pressures, thus obviating the influences of flow conditions and the arterial Windkessel effect. It is important to note that albeit PAWP_*M*_ is designated as a surrogate of diastolic pressures, in fact it comprises the sum of pressure events during both the diastolic and the systolic phase, which indeed distorts the otherwise sound rationale upon which the use of DPG is founded. Recently, our group has demonstrated that the high prevalence of negative DPG can in large part be assigned to the pulsatile LAP components [16] and importantly, might lead to inadequate diagnostic and prognostic assessment. In the present study, roughly half of the patients demonstrated DPG_NEG_ when employing the conventional DPG calculation, whereas PAWP_*Y*_-derived DPG provided a substantial reduction in DPG_NEG_ occurrence. Comparison of the haemodynamic profiles of patients with negative DPG or DPG_*Y*_ values revealed less pronounced haemodynamic alterations in the latter group. Admittedly, despite the significant reduction in DPG_NEG_, these still occurred in 15% of the patients. Even when calculating the DPG by applying PAP_*D*_-synchronized instantaneous PAWP_*S*_ measurements, this approach did not eliminate the occurrence of DPG_NEG_ (20%). This implies that in addition to the influence of pressure pulsatility, other factors, such as catheter whip and/or the limited accuracy of fluid-filled catheters might as well contribute to the occurrence of the incompatible DPG_NEG_ measurements_,_ which remains a limitation in the clinical setting.

Previous studies investigating the functional pulmonary vascular alterations in LHD have focused on patients displaying PH at rest (PAP_*M*_ ≥ 25 mmHg). However, several studies have provided evidence that PAP_*M*_ values close to the upper limit of normal are also associated with long-term increased risk and mortality [30, 31]. Furthermore, due to concomitant diuretic and vasoactive therapy or right heart failure, HF patients often demonstrate normal pressures during resting RHC and the HF-related abnormal haemodynamics might only be evident during exertion [32–35]. Indeed, it has been demonstrated that 20–40% of HF patients undergoing RHC exhibit normal LAP at rest, while abnormal LAP and PAP_*M*_ elevations occur on exertion [34, 36, 37]. This implies that haemodynamic manifestations of pulmonary capillary alterations might occur despite normal PAP_*M*_ at rest. In our cohort, conventional DPG was not significantly different between patients with and without elevated resting PAP and accordingly failed to provide significant prognostic information. In contrast, although the DPG_*Y*_ was lower in patients without PH, it entailed significant prognostic value, even when adjusted for PAP_*M*_ and clinical parameters. Notably, using DPG_*Y*_, 18 patients were reclassified from low- to high-risk group (DPG_*Y*_ ≥ 6 mmHg). Among these patients, the event rate was considerably higher than in the rest of the cohort, indicating that DPG_*Y*_ provides a more sensitive stratification tool compared to conventional DPG. Furthermore, although negative DPG values have been shown to carry generally favourable prognosis [16], adverse events in this group are not infrequent. Importantly, 75% of the DPG_NEG_ patients who experienced an adverse event during follow-up demonstrated positive DPG_*Y*_. The aforementioned results argue for the utility of DPG_*Y*_ as a prognostic marker in HF independently of the presence of PH at rest and suggest that the controversial results regarding the prognostic validity of DPG might in large part reflect shortcomings of the employed methodology rather than the physiologic basis of the DPG index.

## Limitations

The most relevant limitation of the present investigation is the relatively small cohort size. In fact, the lack of prognostic power of conventional DPG after adjustment might partly be due to the relatively limited scale of the study. The fact that the cause of death was not known is also a limitation. Thus, further validation of the suggested method in larger cohorts is warranted. Nonetheless, with the provision of detailed analysis of invasive pressure waveforms, the present report is still among the largest of its kind. Solid catheters provide better accuracy compared to fluid-filled catheters; however, the current approach conforms to the everyday clinical practice, thereby corroborating the clinical impact of our findings. 5% of our original cohort had to be excluded due to inadequate quality of the pressure tracings for reliable PAWP_*Y*_ measurement; however, we believe that carefully recorded, decent tracings are generally a prerequisite to draw appropriate conclusions, independent of the measurement applied. Although the currently employed beat-to-beat haemodynamic analysis might be impractical in the clinical setting, the proposed method may readily be automated thus lending itself for routine use.

## Conclusions

In the present study we show that measuring PAWP at the instantaneous time point of the trough of the *Y*-descent, instead of applying its mean value, results in a significant reduction in the prevalence of negative DPG values. The resulting DPG_*Y*_ demonstrates significant predictive value in heart failure patients, independently of the presence of resting pulmonary hypertension.

## Electronic supplementary material

Below is the link to the electronic supplementary material.

Supplementary file1. Figure S1. Flowchart explaining patient composition of the study cohort. *RHC* right heart catheterisation, *HF* heart failure, *ARVC* arrhythmogenic right ventricular cardiomyopathy, *PH* pulmonary hypertensio. (PDF 72 kb)

Supplementary file2. Figure S2. Prognostic value of onventional DPG in heart failure patients. Kaplan–Meyer curve showing survival of patients with elevated and normal DPG, using cut-off value of 6 mmHg. (PDF 93 kb)
