# Supplementary figures and images for: Optimizing diastolic pressure gradient assessment
Source: Clin Res Cardiol. 2020 May 11;109(11):1411–22. doi: 10.1007/s00392-020-01641-w (PMC7588394; doi:10.1007/s00392-020-01641-w)

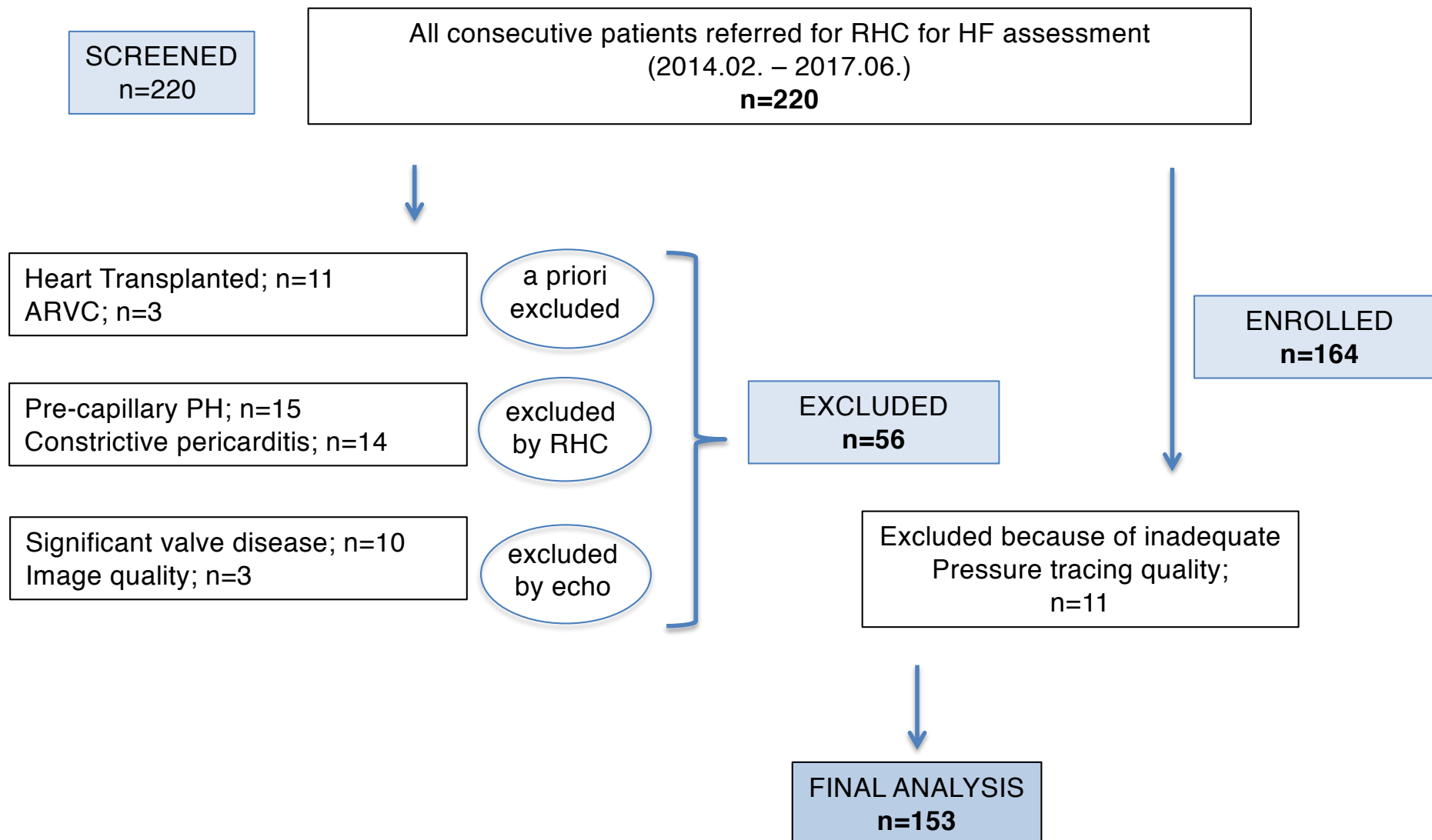

Supplement: Supplementary file 1 — Supplementary file1. Figure S1. Flowchart explaining patient composition of the study cohort. RHC right heart catheterisation, HF heart failure, ARVC arrhythmogenic right ventricular cardiomyopathy, PH pulmonary hypertensio. (PDF 72 kb) [file 392_2020_1641_MOESM1_ESM.pdf]

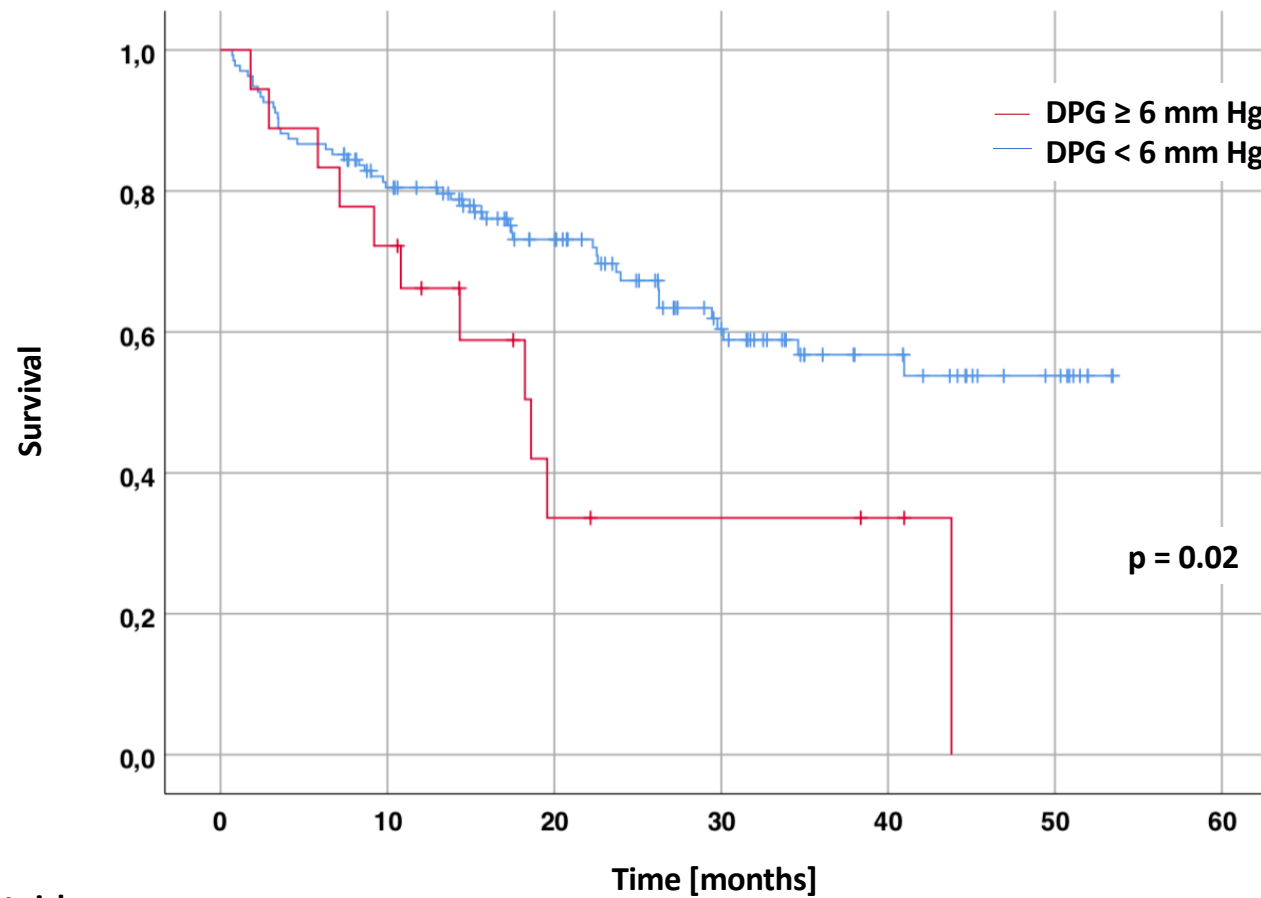

**Number at risk**

|                    |     |     |    |    |    |   |   |
|--------------------|-----|-----|----|----|----|---|---|
| DPG $\geq$ 6 mm Hg | 18  | 13  | 4  | 3  | 2  | 0 | 0 |
| DPG < 6 mm Hg      | 135 | 101 | 71 | 40 | 21 | 9 | 0 |

Supplement: Supplementary file 2 — Supplementary file2. Figure S2. Prognostic value of onventional DPG in heart failure patients. Kaplan–Meyer curve showing survival of patients with elevated and normal DPG, using cut-off value of 6 mmHg. (PDF 93 kb) [file 392_2020_1641_MOESM2_ESM.pdf]
